# Noisy Ocular Recognition Based on Three Convolutional Neural Networks

**DOI:** 10.3390/s17122933

**Published:** 2017-12-17

**Authors:** Min Beom Lee, Hyung Gil Hong, Kang Ryoung Park

**Affiliations:** Division of Electronics and Electrical Engineering, Dongguk University, 30 Pildong-ro 1-gil, Jung-gu, Seoul 100-715, Korea; mblee@dongguk.edu (M.B.L.); hell@dongguk.edu (H.G.H.)

**Keywords:** noisy iris and ocular image, iris and periocular, convolutional neural network

## Abstract

In recent years, the iris recognition system has been gaining increasing acceptance for applications such as access control and smartphone security. When the images of the iris are obtained under unconstrained conditions, an issue of undermined quality is caused by optical and motion blur, off-angle view (the user’s eyes looking somewhere else, not into the front of the camera), specular reflection (SR) and other factors. Such noisy iris images increase intra-individual variations and, as a result, reduce the accuracy of iris recognition. A typical iris recognition system requires a near-infrared (NIR) illuminator along with an NIR camera, which are larger and more expensive than fingerprint recognition equipment. Hence, many studies have proposed methods of using iris images captured by a visible light camera without the need for an additional illuminator. In this research, we propose a new recognition method for noisy iris and ocular images by using one iris and two periocular regions, based on three convolutional neural networks (CNNs). Experiments were conducted by using the noisy iris challenge evaluation-part II (NICE.II) training dataset (selected from the university of Beira iris (UBIRIS).v2 database), mobile iris challenge evaluation (MICHE) database, and institute of automation of Chinese academy of sciences (CASIA)-Iris-Distance database. As a result, the method proposed by this study outperformed previous methods.

## 1. Introduction 

Over the recent years, biometric technology has been widely used in a variety of fields such as financial transactions, access control and smartphone security. Diverse applications of biometric technology have been developed, which can recognize faces, fingerprints, hand geometry, palm prints, irises, retinas, finger-veins, etc. [1,2,3,4]. Iris recognition technology makes use of the pattern of the iris, which has a high degree of freedom (DOF) between the pupil and the sclera [5,6,7]. Iris recognition is finding increasing use in many fields as a contactless recognition technology because of the significant characteristics of irises: iris patterns do not change with age, and even the patterns in the right and left eye of one person are different [7]. When iris images are obtained under unconstrained conditions or without the user’s consent, the quality of the image may be undermined due to optical and motion blur, off-angle view (the user’s eyes looking elsewhere and not into the camera lens), specular reflection (SR) and other factors. These noisy iris images increase intra-individual variations and, as a result, reduce the accuracy of iris recognition. Because more details in the iris texture can be observed by a near-infrared (NIR) illuminator and an NIR camera, the conventional iris recognition system uses this illuminator and camera. However, using the NIR illuminator and an NIR camera makes the system larger and more expensive than fingerprint recognition equipment. An NIR illuminator of high intensity is usually necessary in order to capture the image of clear iris patterns, which leads to additional power consumption and can be an obstacle for the adoption in smartphones. In addition, by capturing the iris image under NIR light, the important information of iris color cannot be used, because the color information cannot be observed under NIR light. To consider these issues with the practical and research reasons, studies have been conducted to investigate iris recognition under visible light.

**Figure 1:**
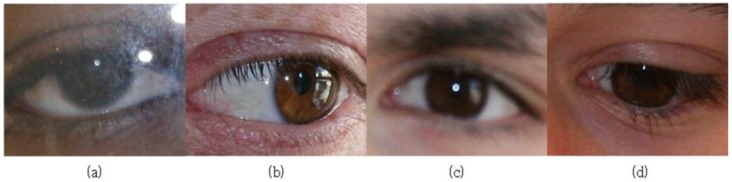
Examples of Noisy Iris Challenge Evaluation-Part II (NICE.II) training dataset. (**a**) Noises by glasses; (**b**) off-angle view and occlusion by ghost area in the right iris region; (**c**) low illumination and blurring; (**d**) occlusion by eyelids and eyelashes.

For example, a study on iris recognition was conducted based on the Noisy Iris Challenge Evaluation-Part II (NICE.II) training dataset [8] extracted from the university of Beira iris (UBIRIS).v2 database. This database includes eye area images deliberately captured from a distance of four to eight meters from the eye under non-constrained conditions to simulate real-life difficulties faced in iris recognition due to poor focus, off-angle view, motion blue, rotation and low illumination. The NICE.II training dataset was used to evaluate the performance of the iris recognition technique through the NICE.II contest [8]. Figure 1 shows some examples of images obtained from the NICE.II training dataset, which are of poor quality because of various factors (low illumination, off-angle view and occlusion by ghost area in the right iris region, blurring, in-plane rotation, noises by glasses, occlusion by eyelids and occlusion by eyelashes).

To consider this issue, this study proposes a new method of ocular recognition by using one iris and two periocular regions based on three convolutional neural networks (CNNs). Our research is novel in the following three ways compared to previous works.

- The method suggested by this study detects only the pupil and iris region without detection of eyelids and eyelashes and SR removal for faster processing speed. By using the three proposed CNNs, we can compensate the accuracy degradation of recognition caused by not detecting eyelids, eyelashes and SR regions.
- This study uses a method of generating one iris image and two periocular images from polar coordinates based on the input iris images. Three pairs of feature vectors are then extracted from three CNNs using one iris image and two periocular images, respectively. A score fusion of the three matching distances obtained from distance matching based on the three extracted pairs of feature vectors is then carried out by applying the weighted product rule. By using the combined score based on three images (one iris image and two periocular images) instead of using one matching score from one image, the accuracy of iris recognition is enhanced, which is experimentally proven.
- In order to make it possible to have fair comparisons by other researchers, we made our trained CNN models public.

## 2. Related Works

In this section, we compare and analyze the relative advantages and disadvantages of various existing iris and periocular recognition methods. There have been various previous studies on iris recognition including iris segmentation, feature extraction and matching [9,10,11,12,13,14]. A study by Dong et al. [14] suggested a method of enhancing the performance of the recognition of individual irises by learning the weight map through intra-class matching of enrolled classes. Rai et al. [15] proposed a method of combining support vector machine (SVM) and hamming distance (HD) with iris recognition. Arora et al. [16] suggested a method of recognition through the decision level fusion (AND rule) of the result of iris recognition of both the eyes irises from a long distance. Shin et al. [17] proposed a method of adaptive bit shifting for matching by applying pre-classification information of the left and right eyes and estimating an in-plane rotation angle based on the position of both eyes, in the process recognizing the irises of both eyes. In addition, smartphones with a system that can recognize the iris of both eyes have been released on the market recently [18]. Park et al. [19,20] studied the periocular region, which refers to the facial area near the eyes, along with the iris, for recognition. Study of the periocular region enables acquisition of images in a relatively less cooperative environment or from farther distances as compared to a study of just the iris. Cho et al. [21] suggested a new periocular recognition method that studies an area with an augmented radius starting from the iris area to include the periocular region based on local binary patterns (LBP) and then converts the dimensions into polar coordinates, which can be adjusted to the rotation of the eyes. He et al. [22] proposed the method of defining a set of data-driven Gabor kernels for fitting the most informative filtering bands by particle swarm optimization (PSO) and binary PSO (BPSO). Then, they acquired the complex pattern from the optimal Gabor filtered coefficients by the deep belief network (DBN). Liu et al. [23] proposed the method of using the convolutional neural network (CNN) with the inputs of two normalized cross-sensor iris images for heterogeneous iris verification.

Baqar et al. [24] proposed the method of using the contour-based feature vector from iris and pupil boundaries as the inputs to DBN with the multilayer feed-forward neural network with variable learning rate (RVLR-NN) for iris recognition. Gangwar et al. [25] proposed the method of using the square-shaped and normalized iris region in the cross-sensor environment as the input to CNN and performing iris recognition by the 4096-feature vector and Euclidean distance. Marra et al. [26] performed the iris sensor model identification by CNN with the iris image. Zhao et al. [27] proposed an accurate and generalizable deep learning method for NIR iris recognition. Their framework is based on a fully-convolutional network (FCN), and a specially-designed extended triplet loss (ETL) function is adopted to incorporate the bit-shifting and non-iris masking, which are found necessary for learning discriminative spatial iris features. They also made a sub-network to provide appropriate information for identifying meaningful iris regions, which serves as essential input for the newly-developed ETL.

Those previous studies on iris recognition were conducted predominantly in an environment of NIR illumination using an NIR camera, which poses a few disadvantages such as large and expensive equipment by using additional NIR illuminators. Due to those disadvantages, recent studies on iris recognition use a general visible-light camera without using an additional NIR illuminator or NIR camera [28]. However, as displayed in Figure 1, iris images captured by a general visible light camera suffer from an inferior definition of the pattern of iris compared to images taken by an NIR camera with NIR illuminator, because of the low level of illumination. In addition, images captured by a general visible light camera can suffer from the ghost effect phenomenon, which refers to visible light reflected from surrounding objects. The ghost effect is more pronounced in the case of visible light cameras as compared to NIR cameras with the NIR illuminator.

Many studies on iris recognition with visible light images, which also take into account the effects of noise, have been conducted based on the NICE.II contest [8,29,30,31,32,33,34,35,36]. Szewczyk et al. [29] suggested a method of performing iris recognition by extracting features based on reverse biorthogonal wavelet transform (RBWT). Li et al. [30] proposed a method for detecting the boundary of irises based on random sample consensus (RANSAC), which can help detect the precise boundary of non-circular irises. A study by De Marsico et al. [31] suggested a method of iris recognition using a combination of the features of LBP and discriminable textons (BLOBs). Li et al. [32] suggested a method of applying weighted co-occurrence phase histograms (WCPH) to iris recognition, for the expression of the local characteristics of texture patterns. A method suggested by Sajjad et al. [33], which was not included in the NICE.II contest, performs iris recognition by using contrast-limited adaptive histogram equalization (CLAHE) [34] on images, in order to be more tolerant to the NICE.II training dataset’s noises such as low contrast and low illumination. A study by Shin et al. [35] proposed a method for performing iris recognition based on SR points of eyelash distribution and lachrymal glands. The proposed method carries out the pre-classification of the left and right eyes, performs the pre-classification of information from RGB channels and different distances of the color space and subsequently performs iris recognition based on the score fusion of the three HDs calculated from the red, green and gray images based on a weighted SUM. A study by Santos et al. [36] proposed a recognition method utilizing periocular information in combination with iris information. A study by Wang et al. [37] proposed a method of performing iris recognition by using adaptive boosting (AdaBoost) training on a multi-orientation 2D Gabor-based feature set. Tan et al. [38] proposed a method for recognition by calculating the matching score based on periocular information, obtaining similarity oriented boosting (SOBoost), and diffusion distance from ordinal measures and color histograms respectively and finally by the fusion of all these scores based on the method by He et al. [39]. Proença et al. [40] proposed the method of combining color and shape descriptors for the recognition of degraded iris images captured by a visible light camera. Tan et al. [41] proposed a method of combining two matching scores by log-Gabor binary features and geometric key encoded features for iris recognition.

As a survey paper, De Marsico et al. [42] explained various previous research works on iris recognition through machine learning techniques including neural network, SVM, fuzzy neural network, PSO, genetic algorithm, etc., leaving the detection and feature extraction issues in the background. As another survey paper, Nguyen et al. [43] showed the state-of-the-art design and implementation of iris-recognition-at-a-distance (IAAD) systems. For that, they explained the design of such a system from both the image acquisition (hardware) and image processing (algorithms) perspectives. In addition, they discussed the significance and applications of IAAD systems in the context of human recognition, provided a review of existing IAAD systems and presented a complete solution to the design problem of an IAAD system, from both hardware and algorithmic perspectives. In addition, they discussed the use of additional ocular information, along with iris, for improving IAAD accuracy, and the current research challenges in addition to presenting recommendations for future research in IAAD.

Many studies have been conducted for iris recognition in noisy visible light environments, but there is room for enhancing the recognition accuracies [38,42,44]. In this paper, we propose a new method of recognition by using one iris and two periocular regions based on the three CNNs. Table 1 depicts a comparison of existing iris recognition methods and the new method proposed by this study.

The subsequent sections of this paper are as follows: Section 3 explains the proposed method suggested by this study in detail, Section 4 provides the results of the experiments conducted, and Section 5 analyzes and discusses these results and ends with a conclusion of the study.

## 3. Proposed Method

### 3.1. Overall Procedure of Proposed Ocular Recognition Method

**Figure 2:**
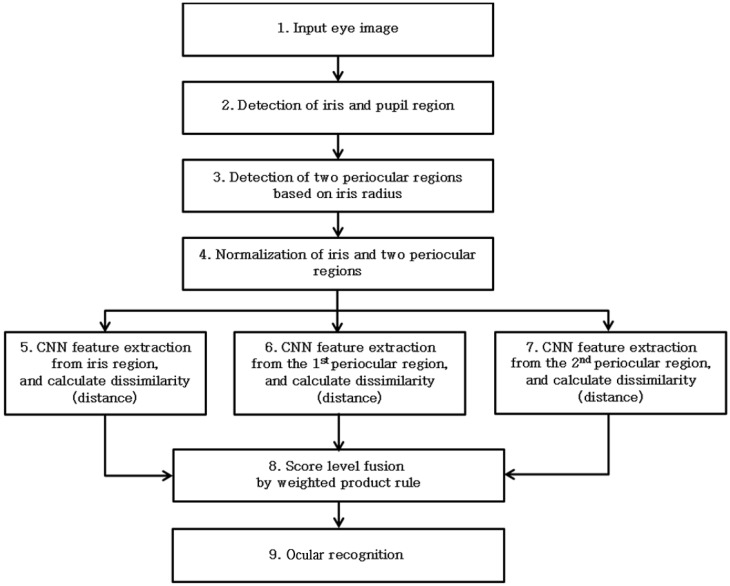
Overview of the proposed method.

Figure 2 displays the overall flow of the algorithm proposed by this study. First, the iris and pupil region is detected from the input eye images (Step (2) of Figure 2). Then, two periocular regions, which are slightly wider than the iris region, are identified based on the detected iris radius (Step (3) of Figure 2). The iris region and the two periocular regions detected in the previous steps are converted into images with polar coordinates, and size normalization is carried out (Step (4) of Figure 2). These images are used as the input for CNN in order to extract the features of CNN, and then, the dissimilarity (distance) between those features and the enrolled features is calculated (Steps (5)–(7) of Figure 2). By conducting the score level fusion of the three dissimilarity values, one score is calculated (Step (8) of Figure 2), and then, ocular recognition (accept as genuine matching or reject as imposter matching) is performed based on the score (Step (9) of Figure 2).

### 3.2. Detection of Iris and Two Periocular Regions

In this section, we explain the method of extracting the iris and the two periocular regions from the input eye images. An accurate segmentation is essential for an accurate iris recognition system that uses a general iris recognition method [45]. Therefore, existing iris recognition algorithms use iris region images from which noise is removed as much as possible through the process of segmentation. In Section 3.2.1, we explain the method of pupil and iris region detection used in this study.

#### 3.2.1. Detection of Iris and Pupil Region

**Figure 3:**
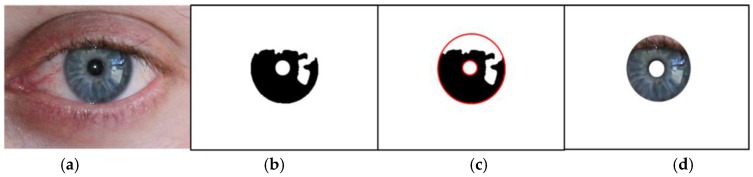
Detection of pupil and iris boundaries. (**a**) Original image; (**b**) binary mask image provided by the NICE.II training dataset; (**c**) results of pupil and iris boundary detection; (**d**) the iris region used in the research.

The NICE.II training dataset used for this study includes images captured by visible light cameras, whose quality is much worse than general iris images taken by NIR cameras with an NIR illuminator. As displayed in Figure 1, it is significantly difficult to detect the iris region from the input images accurately. That is why an international algorithm contest (Noisy Iris Challenge Evaluation-Part I (NICE.I)) for iris region detection by using iris images with such challenges was hosted [46]. The NICE.II training dataset used for this study is the database used in the NICE.II contest. Unlike the NICE.I contest in which the accuracy of iris region detection was evaluated, the NICE.II contest evaluated only the accuracy of the matching algorithm based on iris features [8]. The NICE.II contest utilized Tan et al.’s [47] algorithm, the winning entry of the NICE.I contest, to remove as much noise as possible from the iris binary mask (Figure 3b). Based on the evaluation protocol [29,30,31,32,35,36,37,38] used in NICE.II, the method proposed by this study detects the iris and pupil regions based on the iris binary mask detected by Tan et al.’s [47] algorithm.

However, the iris binary mask provides only the (*x*, *y*) coordinates of each mask pixel, not the information about the center and radius of the pupil and iris. As in general iris recognition methods used in the past, this method also converts the detected iris region into polar coordinate images based on the iris radius calculated using the center and radius of the pupil and iris, as well as the in-plane rotation angle. Therefore, in the method proposed by this study, information about the center and radius of the pupil and iris is obtained by using the following method [35]:

As shown in Figure 3b, the gray value of the iris region is 0, and the gray value of the non-iris region is 255 in binary mask images. First, the geometry center of the zero-value pixel whose gray value is 0 is detected by identifying the rough location of the center of iris. Then, the rough radius of the iris is assumed as half the distance between the leftmost and rightmost pixel of the zero-value pixel. The center and radius of the pupil is determined by applying the following method: First, the white area within the zero-value pixel of the binary mask image is roughly regarded as the pupil region. Then, the geometric center of the region is calculated as the central location of the pupil and a value equivalent to half the distance between the rightmost and leftmost pixel of 255 is set as the radius of the pupil.

Subsequently, based on the rough information about the center and radius of the iris obtained using the aforementioned process, the following circular edge detector (CED) is used in order to find a more accurate center and radius of the iris [35].

(1) $$\underset{\left(x_{0},y_{0}\right),r}{\arg\;\max}\left[\frac{\partial }{\partial r}\left(\int_{-\frac{\pi }{4}}^{\frac{\pi }{6}}\frac{I\left(x,y\right)}{5\pi r/12}ds+\int_{\frac{5\pi }{6}}^{\frac{5\pi }{4}}\frac{I\left(x,y\right)}{5\pi r/12}\;ds\right)\right]$$

where *r* is the radius of the iris region. The coordinates (*x_0_*, *y_0_*) denote the center position of the iris region. By the calculation of the integro-differential operations of Equation (1), the accurate center and radius of the iris region are detected. The two integro-differential operations are done in the range of $-\frac{\pi }{4}\sim \frac{\pi }{6}$ and $\frac{5\pi }{6}\sim \frac{5\pi }{4}$ radians, respectively, as shown in Equation (1). The reason for not performing the operations in the range of 0~2π is because the regions of the other ranges ($-\frac{\pi }{4}\sim \frac{\pi }{6}$ and $\frac{5\pi }{6}\sim \frac{5\pi }{4}$ radians) can be occluded by eyelids, which can degrade the detection accuracy of the iris outer boundary. Figure 3c shows the result of the detected pupil and iris boundaries. As demonstrated in Figure 1, the quality of the iris image captured by the visible light camera used for this study is low, making it difficult to detect the eyelid and eyelash accurately. Incorrect detection of the eyelid and eyelash can cause the consequent degradation of iris recognition. As shown in Figure 3d, iris recognition in this study is performed by using the iris, eyelid and eyelash areas included in the detected center and the radius of the pupil and iris, all of which are used in the CNN.

#### 3.2.2. Detection of Periocular Region Based on Iris Radius and Center Position

**Figure 4:**
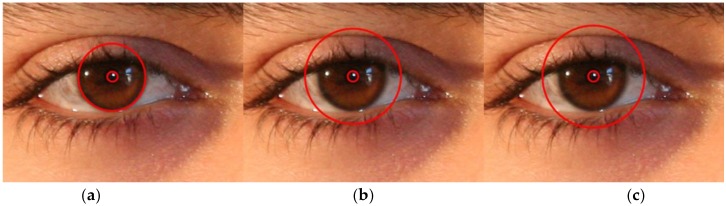
Examples of the iris and periocular regions. (**a**) Iris region; (**b**) periocular region based on *w*_1_ × *IRrad*; (**c**) periocular region based on *w*_2_ × *IRrad*.

As shown in Figure 1, the iris images captured by a visible light camera used for this study are characterized by low distinctiveness of the iris pattern and poor image quality. Therefore, when the method shown in Figure 3d is used for iris recognition, the recognition performance is undermined. In order to resolve this problem, the proposed method augments the image by adding the periocular region to the iris region whose radius (*IRrad*) is augmented based on the central location of the iris. The central location detected using Equation (1) in Section 3.2.1 and the iris region detected from Figure 3d are used in the CNN for iris recognition. In other words, as demonstrated in Figure 4b,c, the region detected as *w*_1_ × *IRrad*, *w*_2_ × *IRrad* (*w*_1_ and *w*_2_ are 1.43 and 1.53, respectively), as well as the iris region are input into the CNN. Here, the optimal *w*_1_ and *w*_2_ were experimentally obtained with training data. In detail, we measured the EER of iris recognition with training data according to the various combinations of *w*_1_ and *w*_2_. From that, we determined the optimal *w*_1_ and *w*_2_, with which the minimum EER of iris recognition was obtained.

#### 3.2.3. Normalization of the Iris and Periocular Region

**Figure 5:**
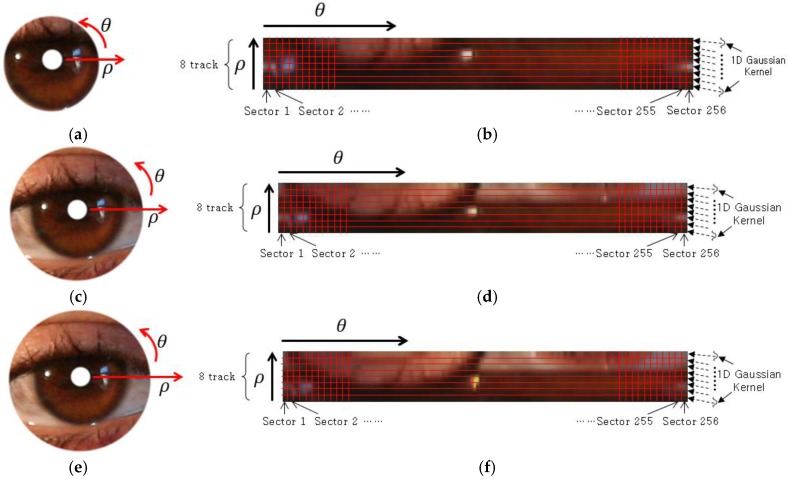
An example of image transformation in polar coordinates and the size normalization. (**a**) Iris region in Cartesian coordinates; (**b**) normalized image of (**a**) in polar coordinates; (**c**) periocular region based on *w*_1_ × *IRrad* in Cartesian coordinate; (**d**) normalized image of (**c**) in polar coordinate; (**e**) periocular region based on *w*_2_ × *IRrad* in Cartesian coordinate; (**f**) normalized image of (**e**) in polar coordinates.

Generally, every person has differently-sized irises. In addition, even for the same person, the size of the iris varies following its dilatation and contraction caused by the change in the level of illumination. In addition, the size of the iris obtained from eye images also becomes different following changes in the distance between the camera and the eyes. Therefore, the following normalization of the iris and periocular images of Figure 4 is performed. In order to cope with the case of inplane rotation of the eye and the change of iris size by external light, the detected iris and periocular regions in Cartesian coordinates (Figure 5a,c,e) are transformed into those of polar coordinates (ρ, θ) for the size normalization [7,35,48]. Then, the iris and periocular images of polar coordinates are respectively divided into eight tracks and 256 sectors as shown in Figure 5b,d,f. In each track, the pixel values are averaged in the vertical (ρ axis) direction by using a one-dimensional (1D) Gaussian kernel. Different from the previous research using two-dimensional Gaussian kernels [5,6,7], we use the one-dimensional Gaussian kernel for computational efficiency. Consequently, the normalized iris and periocular images of 256 × 8 pixels are obtained. The conventional iris recognition algorithm uses the image of eight tracks and 256 sectors [5,6,7]. If the height of the normalized image can be reduced compared to the width as shown in Figure 5, we can also reduce the height of the kernel for the convolutional layer, which can enhance the speed of training and testing of CNN. Therefore, we use the normalized iris and periocular images of 256 × 8 pixels.

### 3.3. Feature Extraction Using Three CNNs

**Figure 6:**
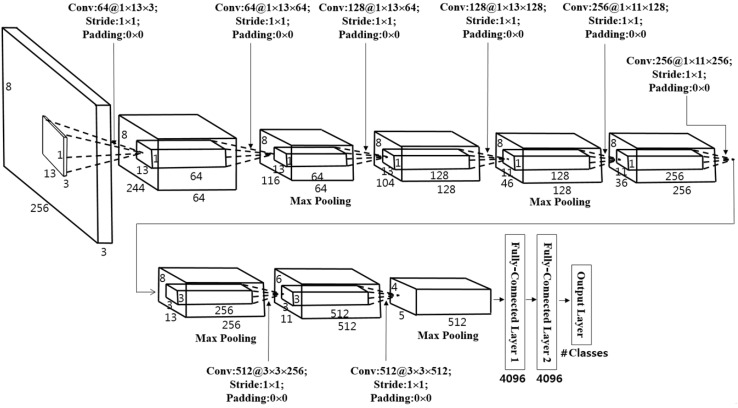
The proposed CNN architecture.

By making use of the normalized iris and periocular images of 256 × 8 pixels explained in Section 3.2.3 and Figure 5, this study used CNN for the feature extraction for iris recognition. Commonly-used existing CNN structures such as AlexNet and visual geometry group (VGG)-Net use square images as input data [49,50]. However, the normalized images used for the iris recognition for this study have 256 pixels in width and 8 pixels in height, so they are not square. In addition, the height is too small to be used in an existing ordinary CNN structure. Therefore, the CNN’s pre-trained filters that are usually square shaped make the convolution of the 256 × 8 pixel images used for this study impossible. In order to resolve such issues, this study proposed a new design of a CNN structure that learns non-square filters appropriate for iris recognition. Table 2 and Figure 6 display the CNN structure designed for this study, which can be described as follows.

In the 1st convolutional layer, 64 filters with a size of 1 × 13 × 3 are used at a stride of 1 × 1 pixels in the horizontal and vertical directions. The size of the feature map is 8 × 244 × 64 in the 1st convolutional layer, such that 8 and 244 are the output height and width, respectively. The calculations are based on: (output width (or height) = (input width (or height) − filter width (or height) + 2 × padding)/stride + 1 [51]). For instance, in Table 2, input width, filter width, padding and stride are 256, 13, 0 and 1, respectively. Therefore, the output height becomes 244 ((256 − 13 + 2 × 0)/1 + 1).

As shown in Table 2, in this study, a 1 × 13 pixel or 1 × 11 pixel non-square filter whose width is longer than its height was used for the 1st–6th convolutional layers. One of the reasons for using such a filter was that an image converted to polar coordinates as demonstrated in Figure 5b,d,f has a width much greater than its height. Another reason was that the vertical (ρ) correlation between patterns is greater than the horizontal (θ) correlation in the iris pattern. Hence, the redundancy of the extracted features can be reduced only when a filter whose width is much longer than its height is used.

Typically, for iris recognition, in-plane rotation occurs in input iris images. In other words, the in-plane rotation (rotation of the iris image in the clock-wise or counter clock-wise direction from the center of the iris) shown in Figure 5a,c,e is an indicator of the horizontal (θ) translation of the images in Figure 5b,d,f, which is a factor for increasing false rejection error (an error of falsely rejecting a person, recognizing him/her as another person). In order to resolve such an issue, preceding studies applied a method of bit shifting in the horizontal direction when performing matching based on the iris binary code extracted by applying the Gabor filter to the normalized iris image of 256 × 8 pixels [7,16,17]. However, in such a case, the imposter matching distribution moves towards the genuine matching distribution, resulting in an issue of increased false acceptance error (an error of falsely accepting a person, recognizing another person as him/her) of iris recognition [7]. However, with a non-square filter, whose width is much longer than its height, such as the one used for this study, such a problem (in-plane rotation of the user’s eye) can be resolved to some extent, without increasing the false acceptance error of iris recognition. That is because the optimal filter coefficients of convolutional layers obtained from CNN training based on the iris images to which various in-plane rotation cases are reflected are tolerant to in-plane rotation to some extent.

Max pooling layers can provide a kind of subsampling. Considering the max pooling layer after the 2nd convolutional layer of Table 2, the feature map of 8 × 232 × 64 is reduced to that of 8 × 116 × 64. Because we used the 1 × 2 × 64 filter with a stride of 1 × 2 for the max pooling layer, the subsampling is performed only in the horizontal (width) direction. Here, 1 × 2 for the number of strides denotes the max pooling filter of 1 × 2 × 64 where the filter moves by two pixels only in the horizontal direction, whereas one pixel in the vertical direction. As demonstrated in Table 2, the number of max pooling layers was decided in consideration of the number of convolution layers. This was decided because not many max pooling layers, which additionally reduce the size of a feature map, were required, since the size of iris and periocular images were already reduced to 256 × 8 pixels during the process of normalization. In other words, the loss of information in images was decreased by reducing the number of max pooling layers, and the filter was applied to as many locations as possible by setting the stride value for filter application (the movement step of the filter) in all convolutional layers at 1.

The batch normalization layer was placed behind the convolution layer, as demonstrated in Table 2. Batch normalization is a method suggested by Ioffe et al. [52], which can accelerate the speed and accuracy of learning. In CNN, the parameters of convolutional layers and the weights of fully-connected layers change in every training step, resulting in an internal covariate shift of the values of input data in every layer. In order to reduce the degree of such shifts in the process of batch normalization, normalization based on the mean value and standard deviation of the values of input data changed in each step was performed and used as the input value for each layer. Through batch normalization, training performance can be enhanced without using the dropout method [49,53], which is generally used for of improving the performance of CNN training. In addition, based on the study by Ioffe et al. [52], this study performed a significant shuffle of the database to select data in each mini-batch randomly for improving the performance of the batch normalization process.

In Table 2, the ReLU layer was used in the form shown in Equation (2) [54,55,56].

(2) y=max(0,x)

where *y* is the output value by the input value (*x*) of the feature map. This function can reduce the vanishing gradient problem [44] that might occur in case a hyperbolic tangent or sigmoid function is used in the training procedure by the back-propagation method, which can result in a better processing speed compared to a non-linear activation function. The ReLU layer exists after each convolutional layer, and it maintains the size of each feature map.

In the third fully-connected layer, the softmax function was used, as shown in Equation (3) below [55].

(3) $$\sigma {\left(s\right)}_{j}=\frac{e^{sj}}{\sum_{n=1}^{K}e^{sn}}$$

Given that the array of output nodes is set to *s*, we could obtain the probability of nodes belonging to the *j*-th class by dividing the value of the *j*-th element by the summation of the values of all the elements.

As explained in the previous studies by Krizhevsky et al. [49], the CNN-based recognition system has an over-fitting problem, which can cause low recognition accuracy with testing data although the accuracy with the training data is still high. To solve this problem, this research employs data augmentation based on the studies by Krizhevsky et al. [49], which can reduce the effects of the over-fitting problem. More details about the outcome of the data augmentation are presented in Section 4.1.

**Figure 7:**
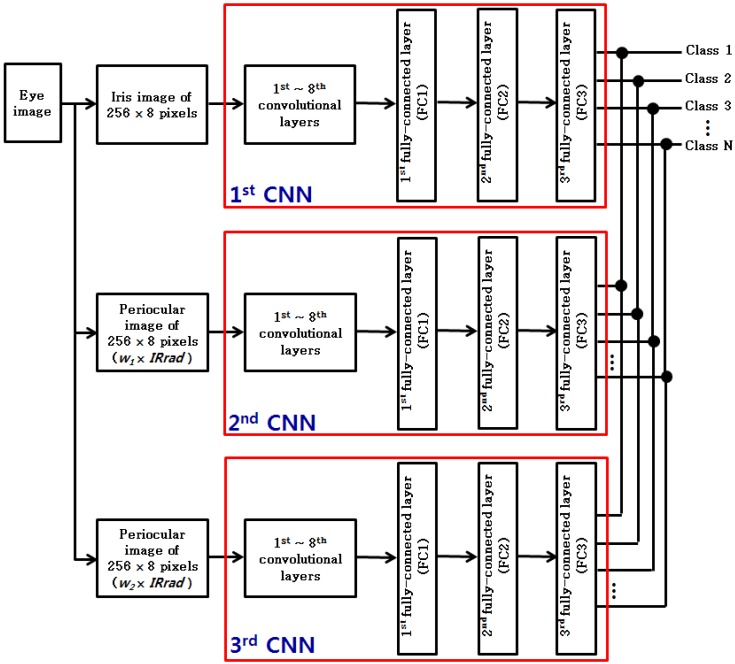
Proposed structure of three CNNs for training and testing.

In this study, each of the three CNNs was trained as demonstrated in Figure 7, by using the three images (iris image of 256 × 8 pixels, periocular image of 256 × 8 pixels (*w*_1_ × *IRrad*) and periocular image of 256 × 8 pixels (*w*_2_ × *IRrad*)) depicted in Figure 5b,d,f. Since the classes of training and the classes of testing are different, the decision information about each class obtained from the third fully-connected layer in Figure 7 cannot be used. Therefore, for the testing of this study, matching was performed by using 4096 features obtained from the first fully-connected layer (FCI) shown in Figure 7. In other words, for testing as well, the three images (iris image of 256 × 8 pixels, periocular image of 256 × 8 pixels (*w*_1_ × *IRrad*) and periocular image of 256 × 8 pixels (*w*_2_ × *IRrad*)) shown in Figure 7 were used as the input for the three trained CNNs (1st–3rd CNNs of Figure 7), respectively. Then, authentic matching and imposter matching were performed by using those three pairs of 4096 features.

### 3.4. Ocular Recognition Based on Score Fusion

In this study, authentic matching and imposter matching were performed using the three pairs of 4096 features explained in Section 3.3. When the Euclidean distance between the three pairs of 4096 features extracted from the enrolled images and the three pairs of 4096 features extracted from the recognized images was calculated, three distances were obtained. Then, matching was performed by using one final distance obtained by combining those three distances based on the score level fusion of weighted sum and weighted product rules. Subsequently, the final distance by score level fusion larger than the threshold was determined as imposter matching and the distance smaller than the threshold as authentic matching. The two errors that occur in the process—false acceptance rate (FAR) (an error of falsely accepting another person) and false rejection rate (FRR) (an error of falsely rejecting a person)—generally have a mutual trade-off relationship. In other words, when FAR grows, FRR declines, and when FAR declines, FRR grows. The error rate at the point when the FAR is the same as FRR is called EER. For this study, the threshold at the point when the EER is obtained was used as the threshold for authentic and imposter matching.

In our research, the optimal rule and weights for weighted sum and weighted product rules were experimentally determined with training data. That is, the rule and weights with which the smallest EER is obtained are finally used as our proposed method.

## 4. Experiment Results

### 4.1. Datasets and Data Augmentation

We used the NICE.II training dataset for conducting the experiments on the method proposed by this study. The NICE.II training dataset was used for the evaluation of the performance in the NICE.II contest [8]. The dataset includes 1000 eye images of 171 classes. The definition of those images is 400 × 300 pixels. The images of the iris were taken of people walking 4–8 m away from a high-resolution visible light camera that uses visible light illumination [57]. Consequently, the images in the dataset exhibited problematic results such as rotation, low illumination, blurring and off-angle view, as well as ghost noise phenomenon, a result of using a visible light camera. Therefore, there exist iris images with low quality in the dataset, as shown in Figure 1.

For learning the CNN model suggested by this study, the number of classes of the dataset was divided by half as displayed in Table 3 to generate two sub-datasets: Group A and Group B. Group A includes 515 images of 86 classes, and Group B includes 485 images of 85 classes. After performing the training of one sub-dataset, testing was conducted on the other sub-dataset (first fold cross-validation) based on the two-fold cross-validation method, and then, the training and testing were conducted once again on the different subsets (second fold cross-validation) to measure average accuracy. In order to make it possible to have fair comparisons by other researchers, we made our trained CNN models public through [58].

As explained in Table 2 and Figure 6, a CNN model that consists of eight convolutional layers and three fully-connected layers was used for this study. The number of images in the training sub-dataset demonstrated in Table 3 was not enough for sufficient learning of the parameters for convolutional layers and weights for fully-connected layers included in the model. The lack of sufficient training data causes the problem of over-fitting, which enables a better classification performance for training datasets, but undermines the performance of the classification for testing datasets [49,53]. In addition, since a variety of noises is included in the NICE.II training dataset used for this study as mentioned above, it was difficult to expect satisfactory classification performance for the testing dataset. In order to address such problems, the volume of the training sub-dataset was increased using data augmentation. Using the *x* and *y* position of the center of pupil, as well as the iris detected by the method of Section 3.2.1, as well as the image translation and redefining method [49] in the original image of Figure 3d, data augmentation was performed. In detail, the center position of the pupil was moved by three positions horizontally and by three positions vertically (3 × 3) in the image of Figure 3d. In addition, the center position of the iris was moved by three positions horizontally and by three positions vertically (3 × 3) in the image of Figure 3d. From that, the normalized images of Figure 5b,d,f were changed. Consequently, the number of images of the original sub-dataset was increased by 81 times by considering the combinations of the number of changes of the pupil and iris center ((3 × 3) × (3 × 3)) for training purposes through data augmentation, as demonstrated in Table 3. For the process of testing, non-augmented original data were used for a fair comparison with the performance in the NICE.II contest. The difference in the center of the pupil and iris region caused by the off-angle view that exists in NICE.II training data, as well as the errors in detecting the iris region, which is caused by rotation, low illumination, blurring and ghost noise, could be compensated by CNN training based on data augmentation.

In this research, CNN training and testing were performed on a system using Intel^®^ Core™ i7-6700 CPU @ 3.4 GHz (four cores) with 32 GB of RAM and NVIDIA GeForce GTX Titan X (3072 CUDA cores) [59] with graphics memory of 12 GB (NVIDIA, Santa Clara, CA, USA).

### 4.2. Training of CNN Model

**Figure 8:**
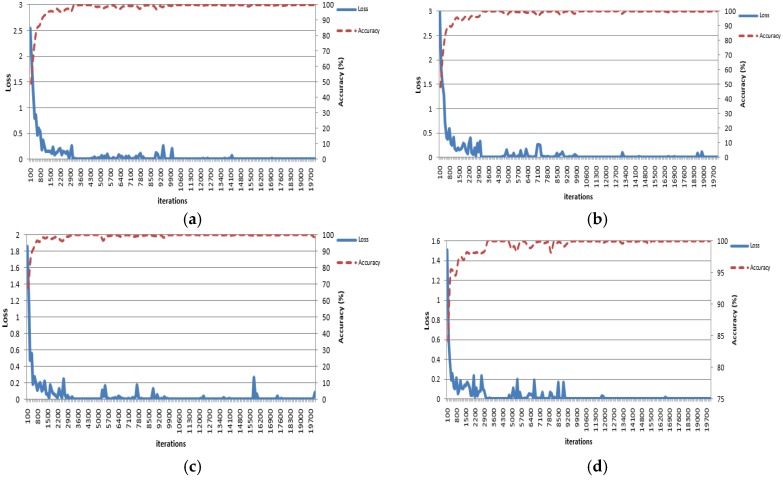

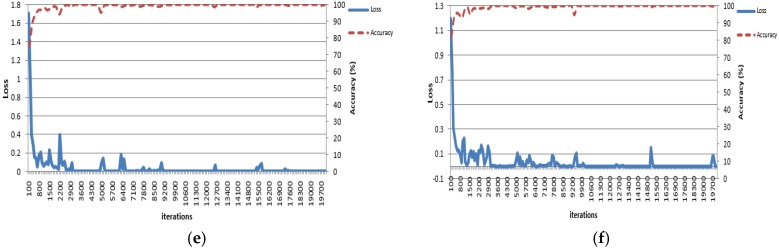
Loss and accuracy curves of CNN training. Using: (**a**) the augmented A sub-dataset for the training of 1st CNN of Figure 7; (**b**) the augmented B sub-dataset for the training of the first CNN of Figure 7; (**c**) the augmented A sub-dataset for the training of the second CNN of Figure 7; (**d**) the augmented B sub-dataset for the training of the second CNN of Figure 7; (**e**) the augmented A sub-dataset for the training of the third CNN of Figure 7; (**f**) the augmented B sub-dataset for the training of the third CNN of Figure 7.

In order to verify the method suggested by this study for two-fold cross-validation, training of the CNN model was conducted by using the training data obtained through the data augmentation explained in Section 4.1. In order to materialize and learn CNN, the Caffe framework [60] was used. In our research, we used the cross-entropy loss function [61] to train the CNN. For the CNN training, we used the Adam optimizer, which is a method used for first-order gradient-based optimization of stochastic objective functions using adaptive estimates of lower-order moments [62]. The initial parameters for this optimizer are, for instance, a learning rate of 0.001, momentum of 0.9, Momentum 2 of 0.999 and epsilon of 1 × 10^−8^, and the detailed explanations of these parameters can be referred to the study by Kingma et al. [62]. The convolution filter was initialized in a method suggested by He et al. [63], and the biases were initialized to zero. A batch size of 128 was used, and learning was conducted in 65 epochs. The volume of the entire training set divided by a mini-batch size was defined as the iteration. The time taken for the complete training including all the iterations was set as one epoch, and the training was performed for the number of times as per a pre-determined epoch. Figure 8 displays Sub-dataset A and Sub-dataset B from Table 3, which shows the training loss and training accuracy when the three CNNs demonstrated in Figure 7 were learned. These kinds of curves of training accuracy and loss represent whether CNN training is successfully performed or not. Therefore, in previous research of CNN-based detection and recognition [49,52,63,64], these graphs have been frequently shown. If training is successfully performed, all training loss approaches zero, and the training accuracy approaches 100%. Based on Figure 8, we can find that our CNN training is successfully performed.

### 4.3. Testing of Proposed CNN-Based Recognition

As mentioned above, the NICE.II training dataset used for this study was used for the performance evaluation in the NICE.II contest [8]. In the NICE.II contest, the performance evaluation was conducted by using the decidability value (*d*-prime value) as depicted in Equation (4).

(4) $$d^{\prime }=\frac{\left|\mu_{A}-\mu_{I}\right|}{\sqrt{\frac{\sigma_{A}^{2}+\sigma_{I}^{2}}{2}}}$$

where $\mu_{A}$ and $\mu_{I}$ denote the means of the authentic and imposter matching distributions, respectively. $\sigma_{A}$ and $\sigma_{I}$ denote the standard deviations of the authentic and imposter matching distributions, respectively. False acceptance cases and false rejection cases take place because of the overlap between authentic and imposter matching distributions. Hence, the farther the two distributions are separated without overlap, the smaller the FAR and FRR are. The *d*-prime value of Equation (4) increases when the two distributions separate and decreases when the overlap increases following the proximity between the two distributions. A greater *d*-prime value is an indicator of a better performance of the biometric system subject to evaluation. For this study as well, the *d*-prime value was used as the benchmark for performance evaluation for an objective assessment of the performance of other methods [29,30,31,32,35,36,37] of the NICE.II contest [8]. In addition, the performance evaluation of this study was also conducted based on EER and the receiver operating characteristic (ROC) curve. As mentioned above, the augmented data were used only for the training process, and non-augmented original data were used for the testing process, for a fair comparison with the performance in the NICE.II contest. Table 4 shows the number of authentic and imposter matching instances during testing.

**Figure 9:**
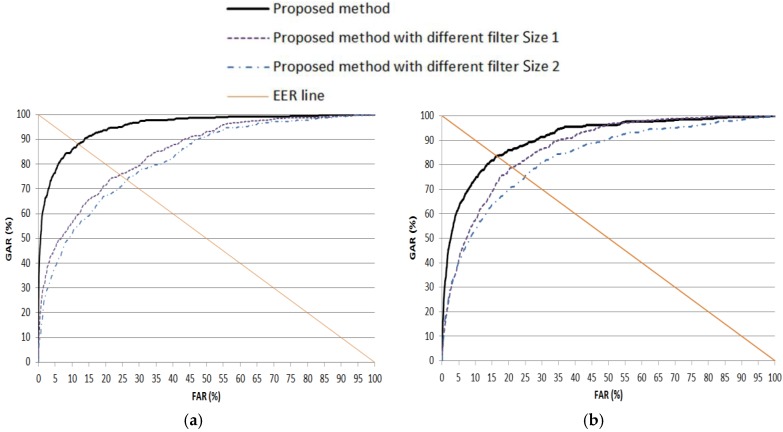
ROC curves of recognition with testing data according to various filter sizes. (**a**) 1st fold cross-validation; (**b**) 2nd fold cross-validation. FAR, false acceptance rate; GAR, genuine acceptance rate.

As the first experiment, we compared the accuracy of iris recognition according to the various filter sizes. As explained in Table 2, our method uses four filters of 1 × 13 and two filters of 1 × 11. As the “different filter Size 1”, we used four filters of 1 × 9 and two filters of 1 × 7. In addition, as the “different filter Size 2”, we used four filters of 1 × 11 and two filters of 1 × 9. The accuracy is measured by the ROC curve, and in Figure 9, the genuine acceptance rate (GAR) is 100 − FRR (%). The experimental result showed that the accuracies of the proposed method using four filters of 1 × 13 and two filters of 1 × 11 were higher than those using other filter sizes.

**Figure 10:**
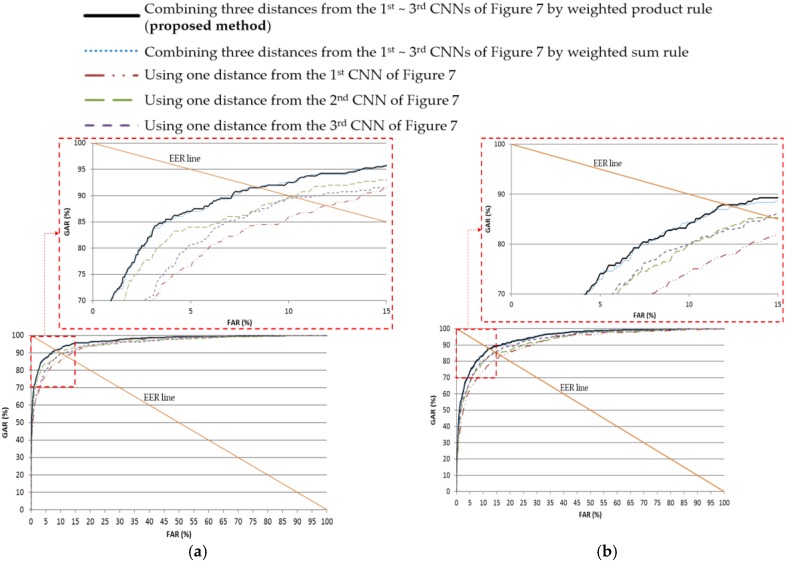
ROC curves of recognition with testing data. (**a**) First fold cross-validation; (**b**) second fold cross-validation.

As the next experiment, in Table 5, the recognition performance of the method suggested by this study and other methods is compared. When recognition was performed with the surrounding periocular region along with the iris region (using one distance from the second CNN of Figure 7 and one distance from the third CNN of Figure 7), the recognition performance was better compared to the recognition performance when only the iris region (using one distance from the first CNN of Figure 7) was used for recognition, as depicted in Table 5. The reason for this difference is that more errors take place for recognition only with the iris region, as the quality of the iris image from the NICE.II training dataset is poorer. In addition, the performance based on the fusion of the weighted product rule used in this study was better than the performance based on a method that applies the score level fusion based on the weighted sum rule. As shown in Figure 10, the accuracy achieved by the proposed method is also higher than by other methods in terms of ROC curves.

We use the image of 256 × 8 (width × height) as the input of CNN as shown in Figure 5b,d,f and Table 2. If we use the filter whose height is not one, because the input height of CNN is as small as eight pixels, multiple convolutional layers with filters whose height is not one cannot be applied without the padding in the vertical (height) direction. Here, the padding in the vertical (height) direction means that the pixel is newly included in the vertical (height) direction of each feature map of Table 2.

As the next experiment, we performed the comparisons of using square filters to our method using non-square filters. In recent CNN models such as VGG-Net [50], VGG-face [65], ResNet [64], etc., a filter of 3 × 3 has been widely used due to its advantage of high processing speed. Based on that, we compared the accuracies by using the filters of 3 × 3 in our CNN architecture of Table 2 to our method. All the other factors including the number of filters, the number of strides, etc., are the same as our CNN architecture of Table 2 for fair comparisons. Only the number of paddings is changed to 2 × 0 (two pixels are newly included in vertical (height) direction of each feature map of Table 2) in this case because the input height of CNN is as small as eight pixels (as shown in Table 2) that convolutional layers with filters of 3 × 3 cannot be applied without the padding of 2 × 0.

Experimental results with the filters of 3 × 3 showed the average EER of 17.35% and average *d*-prime value of 1.77 from two-fold cross-validation, which are worse than those (the average EER of 10.36% and average *d*-prime value of 2.62) by our CNN architecture using non-square filters as shown in Table 5. From these results, we can find the effectiveness of using our non-square filters.

In Table 6, the performance of the recognition method proposed by this study is compared to the performance of other existing methods. As demonstrated by the table, the performance of the recognition method of this study is better than that of other methods in terms of smaller EER and a larger *d*-prime value. Notably, even when compared with the methods of preceding studies [29,30,31,32,33,35,36,37] in the NICE.II contest [8], the performance of this study’s recognition method is better.

### 4.4. Testing with the MICHE Database

**Figure 11:**
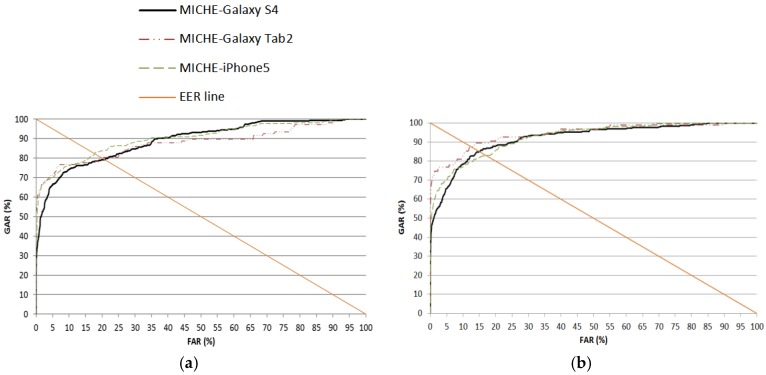
ROC curves of iris recognition in the three sub-datasets of the MICHE database including the data from indoors and outdoors. (**a**) First fold cross-validation; (**b**) second fold cross-validation.

As the next experiment, we compared the accuracies by our method and the previous method with another open database (the MICHE database [66]) of the iris image captured in the indoor and outdoor visible light environments. Like the previous experiments with the NICE.II training dataset in Section 4.1 and Section 4.2, we performed the experiments with the MICHE database based on two-fold cross-validation. Considering the case that a smartphone can usually be used in any place of the indoor and outdoor scenario, we used the images captured indoors and outdoors of the MICHE database for experiments. The EER, *d*-prime value and ROC curve are shown in Table 7 and Figure 11.

Table 8 shows the comparison of the performances by our method with those by previous research. As shown in Table 8, we can find that our method outperforms previous methods. The reason why the accuracies with the MICHE database are lower than those with the NICE.II training dataset as shown in Table 5 and Table 7 is that the image quality of the MICHE database is worse than that of the NICE.II training dataset.

**Figure 12:**
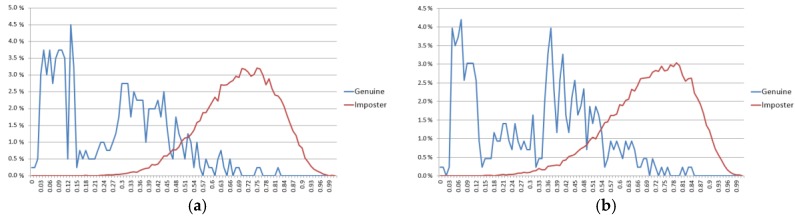
Graphical representations of clients (genuine) and imposters with testing data of the NICE.II dataset. (**a**) First fold cross-validation; (**b**) second fold cross-validation.

In a biometric system, smaller EER and larger *d*-prime values usually represent better accuracies. In Table 6, our method shows higher accuracies (smaller EER and larger *d*-prime value) than all the previous methods. In Table 8, our method also shows smaller EER than all the previous methods and larger *d*-prime values than the previous method by Abate et al. [67]. However, the *d*-prime values by the methods [28,69] are larger than ours. In general, a larger *d*-prime value represents a smaller EER because the *d*-value shows the amount of separability of authentic (client) and imposter distributions as shown in Equation (4). However, because the *d*-prime value is measured based on two means ($\mu_{A}$ and $\mu_{I}$) and standard deviations ($\sigma_{A}$ and $\sigma_{I}$) as shown in Equation (4), if the shapes of two distributions (authentic and imposter distributions) are not similar to a unimodal Gaussian distribution, respectively, the *d*-prime value cannot show the accurate measure of the amount of separability (although the *d*-prime value was used in the NICE.II competition). That is because Equation (4) is based on the assumption of unimodal Gaussian distributions of authentic and imposter distributions [71,72]. Therefore, most research on biometrics uses EER and receiver operational characteristic (ROC) curves for evaluating accuracy more than d-prime. Because most previous research does not show ROC curves in Table 6 and Table 8, we show the accuracies in terms of EER and *d*-prime. As explained, although the *d*-prime values by the methods [28,69] are larger than ours, the EER by our method is smaller than previous methods [28,69], and because the shapes of authentic and imposter distributions are not similar to unimodal Gaussian distributions, we can trust EER more than the *d*-prime value. To prove this, we included the graphical representations of two distributions (clients (genuine)/imposters) of our final result with the NICE.II dataset of Table 5 and Table 6. As explained in Section 4.1, because we measured the EER based on two-fold cross-validation, we show the two graphical representations of two distributions as shown in Figure 12.

**Figure 13:**
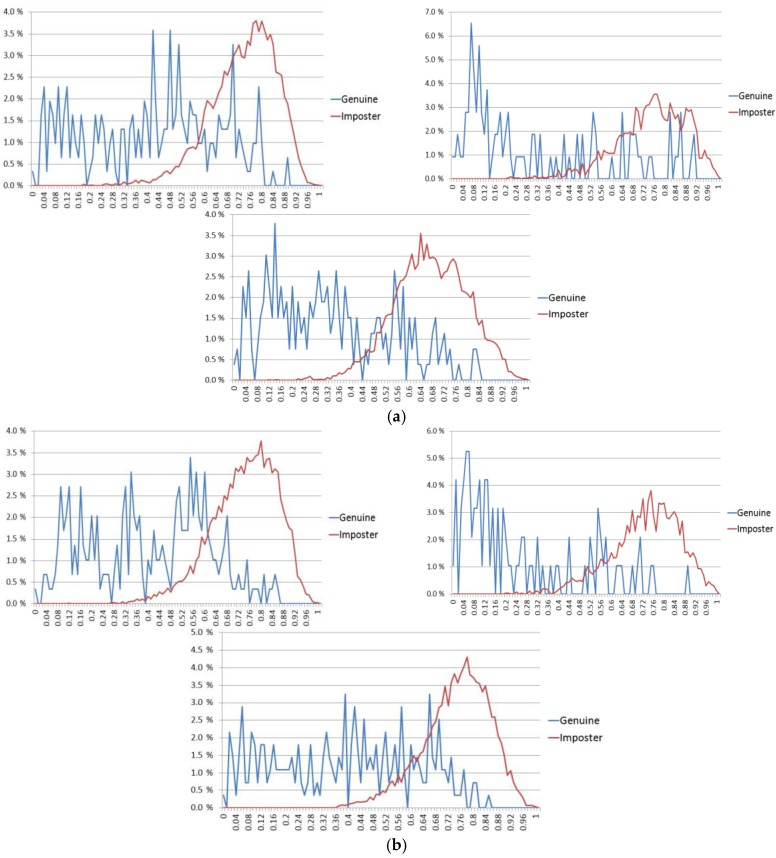
Graphical representations of clients (genuine) and imposters with testing data of the MICHE dataset. (**a**) First fold cross-validation; (**b**) second fold cross-validation. In (**a**) and (**b**), the left-upper, right-upper and center-lower figures respectively show the representations with the sub-datasets of Galaxy S4, Galaxy Tab2 and iPhone5.

In addition, we included the graphical representations of two distributions (clients (genuine)/imposters) of our final result with the MICHE dataset of Table 7 and Table 8. Because the EERs were respectively measured according to the three sub-datasets of Galaxy S4, Galaxy Tab2 and iPhone5 based on two-fold cross-validation, we show the six graphical representations of two distributions as shown in Figure 13.

As shown in Figure 12 and Figure 13, we can find that the shapes of all the representations of two distributions (clients (genuine) and imposters) are not similar to unimodal Gaussian distributions. Especially, the shapes of clients (genuine) are much different from that of the unimodal Gaussian distribution. Therefore, we can trust EER more than the *d*-prime value in our research.

### 4.5. Testing with CASIA NIR Database

As the next experiment, we performed the experiments with the CASIA-Iris-Distance (CASIA-Iris V4) database, which consists of 2567 images obtained from 284 classes of 142 volunteers [73]. This database includes iris images captured by the self-developed long-range multi-modal biometric image acquisition and recognition system (LMBS). Detailed specifications and explanations of the physical system are not unveiled. The magnification factor and focal length of the camera lens are not unveiled, either. The image of this database includes both eyes in a facial image of 2352 × 1728 pixels, and the pixel diameter of the iris area is less than about 170 pixels, which is smaller than other conventional NIR iris databases such as the university of Notre Dame (ND)-IRIS-0405 [74], Indian institute of technology Delhi (IITD) [75] and west Virginia university (WVU) Non-ideal databases [76]. In addition, in order to consider the various capturing environments along the long Z-distance (from a distance of 2.4–3 m [73], various noise factors such as severe off-angle, specular reflection on glasses, low illumination and hair occlusion were included in the CASIA-Iris-Distance database. Therefore, the accuracy of iris recognition with CASIA-Iris-Distance is usually lower than those with other NIR iris databases [27].

With this database, we compared our accuracy with those by previous methods [17,27,77]. For fair comparisons, we performed the experiments based on the experimental protocols of [17,27,77].

Because Shin et al. [17] used four-fold cross-validations, we measured the accuracies based on four-fold cross-validations of same data. Experimental results showed that the EERs from the 1st–4th fold cross-validations by our method were 2.98%, 2.87%, 3.26 and 3.03%, respectively. Average EER was 3.04%.

As the second experiment, we used the same protocol of Zhao et al.’s method [27] and performed the experiments, again. As explained in Zhao et al.’s method [27], the training set consists of all the right eye images from all the subjects, and the test set comprises all the left eye images. Based on this protocol, in the first fold cross-validation of our experiments, we used all the right eye images from all the subjects as the training set, whereas all the left eye images from all the subjects were used as the testing set (same protocol to Zhao et al.’s method [27]). In the second fold cross-validation, we exchanged the training and testing sets with each other and repeated the experiment. Experimental results showed that the EER of the first fold cross-validation (the same protocol to Zhao et al.’s method [27]) was 3.05%, whereas that of the second fold cross-validation was 3.14%. From that, the average EER was 3.10%.

As the third experiment, we performed the training and testing again based on the experimental protocols of Sharifi et al.’s method [77]. In this research, the whole database was divided into two independent sets called Set-I (validation set) and Set-II (testing set). Set-I consisted of 52 subjects, and each subject possessed 10 images. In Set-II, 90 subjects were included, and each subject possessed 10 samples. Specifically, Set-II was divided into two equal partitions presenting five reference and five testing data for all the subjects in the database. The partitioning of these two subsets (reference and testing) was performed 10 times without any overlapping. The average accuracies of 10 times was measured as the final accuracy. Based on this protocol, we performed the experiments again. Experimental results showed that the EERs from the 1st–10th trials were respectively 3.14%, 3.12%, 2.98%, 3.05%, 3.24%, 2.87%, 2.92%, 3.05%, 3.21% and 3.19%. From that, the average EER was 3.08%.

From these results, we can find that the errors by our method were lower than those by previous methods (EER of 4.30% by Shin et al. [17], EER of 3.85% by Zhao et al. [27] and EER of 3.29% by Sharifi et al. [77]), as shown in Table 9, and the effectiveness of our method even with the NIR iris database is confirmed.

### 4.6. Processing Time

As the last experiment, we measured the average processing time per each image of the NICE.II database. The experimental results showed that the average processing time of each image was about 96.4 ms, which includes the time of 96 ms (from the Step (2)–Step (7) in Figure 2) and that of 0.4 ms (for the Steps (8) and (9) in Figure 2). From that, we can find that our system can be operated at a speed of about 10.4 frames per second (1000/96.4). Processing time was measured on a system using Intel^®^ Core™ i7-6700 CPU @ 3.4 GHz (four cores) with 32 GB of RAM and NVIDIA GeForce GTX Titan X (3072 CUDA cores) [59] with graphics memory of 12 GB (NVIDIA, Santa Clara, CA, USA). The processing time per each image of the MICHE database and CASIA-Iris-Distance was similar to that of NICE.II database, which were 94.2 ms and 92.8 ms, respectively.

## 5. Conclusions

This study suggested a new method for iris recognition based on CNN. By using three CNNs designed to be appropriate for normalized iris and periocular images of a size of 256 by eight pixels, three distances based on three pairs of features obtained from each CNN were combined by applying a weighted product rule to perform authentic and imposter matching. Our recognition performances in two-fold cross-validation, using the NICE.II training dataset, MICHE database and CASIA-Iris-Distance database were found to be better than the methods suggested by other studies.

In future work, we will study the method to enhance the recognition accuracies by using a deeper CNN structure. In addition, we will research the method to combine both deep features by our CNN with conventional features by Gabor filtering or other kinds of hand-crafted features.

## Figures and Tables

**Table 1:** Comparison of existing recognition methods and the new method proposed by this study (*d’* means the *d*-prime value of Equation (4). EER means equal error rate, and its concept is explained in Section 3.4). LBP, local binary patterns; RBWT, reverse biorthogonal wavelet transform; WCPH, weighted co-occurrence phase histograms; CLAHE, contrast-limited adaptive histogram equalization.

Category	Method	Periocular Region	Accuracy	Advantage	Dis-Advantage
NIR camera- based	Personalized weight map [14]	Not using	EER of 0.78% (**A**)	Better image quality and recognition performance than the visible-light camera method	- Large and expensive NIR illuminator with NIR camera - Additional power usage by NIR illuminator
	SVM with hamming distance (HD) [15]		EER of 0.09% (**B**)		
			EER of 0.12% (**C**)		
	Fusion (AND rule) of left and right irises [16]		Accurate EER is not reported (EER of about 18–21% (**D**))		
	Adaptive bit shifting for matching by in-plane rotation angles [17]		EER of 4.30% (**D**)		
	LBP with iris and periocular image in polar coordinate [21]	Using	EER of 10.02% (**D**)		
	Log-Gabor binary features with geometric key encoded features [41]	Not using	EER of 19.87%, d’ of 1.72 (**E**)	Same algorithm for NIR and visible light iris images	Using manual hand-crafted features
			EER of 3.56%, d’ of 5.32 (**D**)		
	CNN-based method (**Proposed method**)	Using	EER of 3.04–3.10% (**D**)		Intensive CNN training is necessary
Visible light camera- based	Log-Gabor binary features with geometric key encoded features [41]	Not using	EER of 16.67%, d’ of 2.08 (**F**)		Using manual hand-crafted features
	RBWT [29]		d’ of 1.09 (**G**)	Recognition is possible with general visible-light camera without additional NIR illuminator	- Image brightness is affected by environ- mental light - Greater ghost effect caused by reflected light from environ- mental objects
	Non-circular iris detection based on RANSAC [30]		d’ of 1.32 (**G**)		
	Fusion of LBP and BLOBs features [31]		d’ of 1.48 (**G**)		
	WCPH-based representation of local texture pattern [32]		d’ of 1.58 (**G**)		
	CLAHE-based image enhancement [34]		EER of 18.82% (**G**)		
	Pre-classification based on eyes and color [35]		EER of 16.94%, d’ of 1.64 (**G**)		
	LBP-based periocular recognition [36]	Using	EER of 18.48%, d’ of 1.74 (**G**)		
	AdaBoost training by multi-orient 2D Gabor feature [37]	Not using	d’ of 2.28 (**G**)		
	Combining color and shape descriptors [40]		EER of about 16%, d’ of about 2.42 (**G**)		
	CNN-based method (**Proposed method**)	Using	EER of 10.36%, d’ of 2.62 (**G**)	Same algorithm for NIR and visible light iris images	Intensive CNN training is necessary
			EER of 16.25–17.9%, d’ of 1.87–2.26 (**H**)		

**A**: Institute of automation of Chinese academy of sciences (CAISA)-IrisV3-Lamp database; **B**: CASIA-Iris-Ver.1 database; **C**: Chek database; **D**: CASIA-Iris-distance database; **E**: Face recognition grand challenge (FRGC) database; **F**: University of Beira iris (UBIRIS).v2 database; **G**: NICE.II training dataset; **H**: Mobile iris challenge evaluation (MICHE) database.

**Table 2:** The proposed CNN architecture used in our research (ReLU means rectified linear unit).

Layer Type	Number of Filters	Size of Feature Map (Height × Width × Channel)	Kernel (Filter) Size (Height × Width × Channel)	Number of Stride (Height × Width)	Number of Padding (Height × Width)
Image input layer		8 × 256 × 3			
1st convolutional layer	64	8 × 244 × 64	1 × 13 × 3	1 × 1	0 × 0
Batch normalization		8 × 244 × 64			
ReLU layer		8 × 244 × 64			
2nd convolutional layer	64	8 × 232 × 64	1 × 13 × 64	1 × 1	0 × 0
Batch normalization		8 × 232 × 64			
ReLU layer		8 × 232 × 64			
Max pooling layer	1	8 × 116 × 64	1 × 2 × 64	1 × 2	0 × 0
3rd convolutional layer	128	8 × 104 × 128	1 × 13 × 64	1 × 1	0 × 0
Batch normalization		8 × 104 × 128			
ReLU layer		8 × 104 × 128			
4th convolutional layer	128	8 × 92 × 128	1 × 13 × 128	1 × 1	0 × 0
Batch normalization		8 × 92 × 128			
ReLU layer		8 × 92 × 128			
Max pooling layer	1	8 × 46 × 128	1 × 2 × 128	1 × 2	0 × 0
5th convolutional layer	256	8 × 36 × 256	1 × 11 × 128	1 × 1	0 × 0
Batch normalization		8 × 36 × 256			
ReLU layer		8 × 36 × 256			
6th convolutional layer	256	8 × 26 ×256	1 × 11 × 256	1 × 1	0 × 0
Batch normalization		8 × 26 × 256			
ReLU layer		8 × 26 × 256			
Max pooling layer	1	8 × 13 × 256	1 × 2 × 256	1 × 2	0 × 0
7th convolutional layer	512	6 × 11 × 512	3 × 3 × 256	1 × 1	0 × 0
Batch normalization		6 × 11 × 512			
ReLU layer		6 × 11 × 512			
8th convolutional layer	512	4 × 9 × 512	3 × 3 × 512	1 × 1	0 × 0
Batch normalization		4 × 9 × 512			
ReLU layer		4 × 9 × 512			
Max pooling layer	1	4 × 5 × 512	1 × 2 × 512	1 × 2	0 × 1
1st fully connected layer		4096			
Batch normalization		4096			
ReLU layer		4096			
2nd fully connected layer		4096			
Batch normalization		4096			
ReLU layer		4096			
3rd fully connected layer		# of classes			
Softmax layer		# of classes			
Classification layer (output layer)		# of classes			

**Table 3:** Description of the experimental dataset.

Dataset Group	Number of Class	Number of Image before Data Augmentation	Number of Image after Data Augmentation
NICE.II training dataset	171	1000	81,000
A sub-dataset	86	515	41,715
B sub-dataset	85	485	39,285

**Table 4:** Number of intra-class and inter-class for each dataset group.

Dataset Group	Number of Class	Number of Authentic Matching Instances	Number of Imposter Matching Instances
A sub-dataset	86	429	36,465
B sub-dataset	85	400	33,600

**Table 5:** Comparisons of recognition accuracies.

Method	Two-Fold Cross Validation	EER (%)		*d*-Prime Value	
		1st and 2nd Fold	Average	1st and 2nd Fold	Average
Using one distance from the 1st CNN of Figure 7	1st fold	11.86	14.16	2.49	2.24
	2nd fold	16.45		1.99	
Using one distance from the 2nd CNN of Figure 7	1st fold	10.16	12.42	2.61	2.36
	2nd fold	14.67		2.12	
Using one distance from the 3rd CNN of Figure 7	1st fold	10.27	12.39	2.53	2.36
	2nd fold	14.51		2.18	
Combining three distances from the 1st–3rd CNNs of Figure 7 by weighted sum rule	1st fold	8.58	10.63	2.86	2.61
	2nd fold	12.69		2.36	
Combining three distances from the 1st–3rd CNNs of Figure 7 by weighted product rule (**proposed method**)	1st fold	8.46	**10.36**	2.87	**2.62**
	2nd fold	12.26		2.38	

**Table 6:** Comparison between the recognition method suggested by this study and methods of other preceding studies (*N.R.* means, “not reported”).

Method	EER (%)	*d*-Prime Value
Szewczyk et al.’s [29] method	*N.R.*	1.09
Li et al.’s [30] method		1.32
De Marsico et al.’s [31] method	25.8 * (approximate value)	1.48
Li et al.’s [32] method	*N.R.*	1.58
Sajjad et al.’s [33] method	18.82	*N.R.*
Shin et al.’s [35] method	16.94	1.64
Santos et al.’s [36] method	18.48	1.74
Wang et al.’s [37] method	19.1 ** (approximate value)	2.28
Proença et al.’s [40] method	16 (approximate value)	2.42 (approximate value)
Tan et al.’s [38] method	12 *** (approximate value)	2.57
**Proposed method**	**10.36**	**2.62**

*, ** and ***: reported in the study by Proença et al. [40].

**Table 7:** EER and *d*-prime value of iris recognition in the three sub-datasets of the MICHE database including the data from indoors and outdoors.

Sub-Dataset	Two-Fold Cross Validation	EER (%)		*d*-Prime Value	
		1st and 2nd Fold	Average	1st and 2nd Fold	Average
Galaxy S4	1st fold	20.79	17.9	1.76	1.87
	2nd fold	15.01		1.98	
Galaxy Tab2	1st fold	19.89	16.25	1.95	2.26
	2nd fold	12.6		2.56	
iPhone5	1st fold	17.64	17.45	1.96	2.00
	2nd fold	17.26		2.03	

**Table 8:** Comparison between the recognition method suggested by this study and the methods of other preceding studies with the MICHE database including the data from indoors and outdoors (*N.R.* means “not reported”).

Method	Sub-Dataset	EER (%)	*d*-Prime Value
Abate et al.’s [67] method	Galaxy S4	36.7	0.65
	Galaxy Tab2	39.1	0.60
	iPhone5	39.9	0.51
Barra et al.’s [68] method	Galaxy S4	45 (approximate value)	*N.R.*
	Galaxy Tab2	46 (approximate value)	
	iPhone5	43 (approximate value)	
Raja et al.’s [28] method *	Galaxy S4	38.8	6.49
	Galaxy Tab2	33.9	8.63
	iPhone5	38.6	6.21
Santos et al.’s [69] method **	Galaxy S4	19.8	6.13
	Galaxy Tab2	16.3	6.20
	iPhone5	22	5.44
**Proposed method**	Galaxy S4	17.9	1.87
	Galaxy Tab2	16.25	2.26
	iPhone5	17.45	2.00

* and **: the accuracies are reported in the study by De Marsico et al. [70].

**Table 9:** Comparison between the recognition method suggested by this study and the methods of other preceding studies with the CASIA-Iris-Distance database.

Method	EER (%)
Method by Shin et al. [17]	4.30
Proposed method based on the experimental protocol of shin et al. [17]	3.04
Method by Zhao et al. [27]	3.85.
Proposed method based on the experimental protocol of Zhao et al. [27]	3.05
Method by Sharifi et al. [77]	3.29
Proposed method based on the experimental protocol of Sharifi et al. [77]	3.08
